# Expression of *AhDREB1*, an AP2/ERF Transcription Factor Gene from Peanut, Is Affected by Histone Acetylation and Increases Abscisic Acid Sensitivity and Tolerance to Osmotic Stress in *Arabidopsis*

**DOI:** 10.3390/ijms19051441

**Published:** 2018-05-11

**Authors:** Baihong Zhang, Liangchen Su, Bo Hu, Ling Li

**Affiliations:** 1 Guangdong Provincial Key Laboratory of Biotechnology for Plant Development, School of Life Sciences, South China Normal University, Guangzhou 510631, China; zhangbyhome@126.com (B.Z.); sksky163@163.com (L.S.); hubo0610@126.com (B.H.); 2 Department of Bioengineering, Zhuhai Campus of Zunyi Medical University, Zhuhai 519041, China

**Keywords:** APETALA2/Ethylene Responsive Factor, *AhDREB1*, peanut, abscisic acid, drought stresses, histone acetylation

## Abstract

Drought stress negatively affects plant growth and development. An increasing number of reports have revealed the involvement of APETALA2/Ethylene Responsive Factor (AP2/ERF) transcription factors (TFs) in biotic and abiotic stress regulation in plants. However, research on these TFs in the peanut plant *(Arachis hypogaea)* has been limited. Here, we isolated a full-length coding sequence (CDS) of the AP2/ERF family gene *AhDREB1* from the peanut plant and showed that its expression was induced by Polyethylene Glycol (PEG) 6000 and exogenous abscisic acid (ABA) treatment. When overexpressed in *Arabidopsis*, *AhDREB1* increased both ABA levels and ABA sensitivity, affected the ABA signaling pathway and increased the expression of downstream drought stress-related genes *RD29A*, *P5CS1*, *P5CS2* and *NCED1*. These results demonstrate that *AhDREB1* can improve tolerance to drought via the ABA-dependent pathway in *Arabidopsis*. In the peanut plant, the specific histone deacetylases (HDACs) inhibitor trichostatin A (TSA) promotes *AhDREB1* transcription and the enrichment level of H3ac was increased in regions of the *AhDREB1* gene during TSA and PEG treatment. In summary, histone acetylation can affect the expression of *AhDREB1* under osmotic stress conditions, thereby improving plant drought resistance.

## 1. Introduction

Plants are sessile and constantly subjected to various abiotic stresses, including drought, cold, high salinity, heat and alkalinity, which seriously affect their growth, development and productivity [1]. Of these stresses, drought is one of main causes of loss in production of agricultural crops across the world [2]. Drought can result in plant water loss and leaf wilting, and can lead to irreversible, damaging changes including, ultimately, plant death [3].

Over a long period of evolution, plants have developed diverse survival strategies and complex mechanisms to cope with drought stress [4]. Plants respond to drought stress by altering molecular and physiological processes at the molecular, cellular, tissue and whole-plant levels [5,6]. At the molecular level, plants can optimize their growth and development by regulating the expression of numerous stress responses via two main pathways, one of which is abscisic acid (ABA)-dependent, while the other is not [7]. However, several studies suggest that, in fact, there may be cross-talk between the two types of signaling pathway; thus, they may be interdependent [8]. The phytohormone ABA is a pivotal regulator of plant growth and development and of plant responses to drought stress [3]. The content of ABA in plants increases rapidly under drought stress conditions, and returns to its original level when the stress is removed. Because it correlates with drought resistance, the level of ABA is an important index of drought resistance in plants [9]. Multiple processes are involved in the biosynthesis of ABA, but the main factor during drought is the increase in expression of *9-cis-epoxycarotenoid dioxygenase (NCED)*, which encodes a key enzyme in the ABA biosynthetic pathway [10,11]. In addition, plants regulate the expression of drought-resistance genes via relevant components of ABA signaling pathway, thus protecting plants from drought stress. ABA can change the transcription level of drought-related genes via the ABA-responsive element (ABRE) binding protein [12,13].

In the ABA-independent pathway, dehydration-responsive element binding protein (DREB), plays the main role in regulating many drought-responsive genes. Other transcription factors such as NAC (NAM, ATAF, CUC), MYB (v-myb avian myeloblastosis viral oncogene homolog), MYC (Myelocytomatosis proteins), WRKY and nuclear factor-Y (NF-Y) are also involved in drought response and tolerance [14]. DREB activates drought-associated genes, such as *RD29A*, *P5CS1*, *SODs*, by binding to dehydration responsive element (DRE) motifs [15,16], which comprise nine conserved bases (TACCGACAT), in their promoter regions, thereby increasing the plant’s drought resistance [14,17]. DREB transcription factors form a subfamily of the APETALA2/Ethylene Responsive Factor (AP2/ERF) protein family [18,19].

AP2/ERFs are a large family of plant-specific transcription factors (TFs) and the rapid development of genome-wide sequencing technology and bioinformatics tools has led to the identification of AP2/ERF genes in many species; for example, 139 and 122 AP2/ERF genes have been found in rice and *Arabidopsis thaliana*, respectively [20]. TFs from the AP2/ERF family have either one or two highly conserved DNA-binding AP2/ERF domains, each of 58 or 59 amino acids residues [21]. Based on DNA binding domain (DBD) and sequence similarity, AP2/ERF family members have been divided into four major subfamilies, comprising DREB factors, ERFs, AP2 factors and a subfamily called Related to ABI3 and VP1 (RAV) [18,19]. The DREB and ERF subfamilies, both containing a single AP2 domain, are distinguished from each other by differences in the conserved residues of their DBDs. In DREB proteins, the 14th and 19th positions of the DBD are valine (V14) and glutamic acid (E19), whereas in ERFs, the corresponding positions are alanine (A14) and aspartic acid (D19) [18]. Members of the AP2 subfamily have two AP2/ERF domains, while members of the RAV subfamily have a B3 DBD in addition to an AP2/ERF domain [18,19].

In recent years, an increasing number of reports have revealed AP2/ERF TFs to be involved in multiple biological processes that regulate plant growth and development, and especially in abiotic stress tolerance. Among the AP2/ERF family, DREB and ERF subfamily proteins have been well characterized in abiotic stress responses involving drought, salt, cold and other stresses [20]. Thus, overexpression of *GsERF71*, which encodes an AP2/ERF TF from wild soybean, improves plant alkaline stress tolerance by upregulating H^+^-ATPase and by modifying the accumulation of auxin [22]. In *Arabidopsis*, overexpressing *AtERF019* increases tolerance to water deficiency, and also results in phenotypes that delay flowering and senescence [23]. In *Arabidopsis*, overexpression of *DREB1* can enhance tolerance to cold stress, promote the accumulation of proline and result in phenotypes of plant dwarfing and delay of flowering [24]. Overexpression of *ScDREB8* significantly improves salt tolerance of *Arabidopsis* at the seedling stage by upregulating the expression of downstream stress-related genes and improving reactive oxygen species (ROS) scavenging ability [25]. Overexpression of *OsDREB* genes enhances drought tolerance in rice [26]. In *Arabidopsis*, strains overexpressing *AtDREB2C* are highly sensitive to ABA during seed germination and show enhanced tolerance to cold and heat stress [8].

It has been shown that epigenetic regulation plays an important role in plant growth and development [27]. Epigenetic regulation mainly involves two aspects of DNA and histone. At the level of DNA, it is mainly the methylation of genomic DNA, while at the histone levels are mainly histone acetylation, methylation, glycosylation, phosphorylation, ubiquitination, and so on [28,29]. Histone acetylation levels are controlled by the dynamic equilibrium between histone acetyltransferases (HATs) and histone deacetylases (HDACs) [30]. Thus, acetylation of histone lysine residues by HATs can relax chromatin structure and thus facilitate gene activation. Conversely, HDACs remove acetyl groups from acetylated histones, and this activity is generally associated with transcriptional repression and gene silencing [31]. Recently, some studies have shown that histone acetylation can affect the development of plant seeds, the formation and opening of flowers, the elongation of roots, gametophyte development and other biological processes. [32,33]. In maize, mutation of the HDAC gene *hda101* results in a smaller kernel than that of wild type, and in defects of basal endosperm transfer layer (BETL) cells, which suggests that HDA101 can affect the development of seed in maize [34]. In *A. thaliana*, *AtHDA5* affects flowering time, and the *hda5* mutant exhibits a late-flowering phenotype [35]. An increasing number of reports have shown that histone acetylation affects ABA and other phytohormone signaling pathways, and is also important for the epigenetic regulation of plant responses to external stress [36,37]. However, the specific mechanisms are not clear. Recently, it has been reported that the MSI1-HDA19 complex affects ABA signaling pathway by binding to the chromatin of ABA receptor genes *PYL2*, *PYL4* and *PYL6*, where it maintains low levels of acetylation of histone H3 at lysine 9, thereby regulating expression levels of these genes [38]. In *A. thaliana*, AtHDA9 negatively affects plant sensitivity to drought and salt stresses by modifying histone acetylation levels of a large number of stress-responsive genes [27]. In maize, HDACs can affect the level of histone acetylation in chromatin, which in turn promotes the expression of *ZmDREB1* under cold stress [39].

Peanut (*Arachis hypogaea* L.) is one of the most important oil crops and food sources of protein, and is cultivated in the semi-arid tropical and subtropical regions of the world [40]. Peanut plants are constantly subjected to various biotic and abiotic stresses during growth and development. Drought stress is one of the main factors that limit the growth, yield and quality of peanuts [36]. Therefore, it is meaningful to study how peanut plants respond to drought stress, which has potential applications in the breeding of drought-resistant strains. Previously, in the peanut transcriptome database, we found a *DREB-like* gene (*comp63385_c0*) that contained a key AP2/ERF domain and was upregulated by water deficit and exogenous ABA [41]. This gene was named *AhDREB1* (GenBank accession No. KU143745.1). In this paper, the full-length coding sequence (CDS) of *AhDREB1* was isolated from peanut leaves and its transcription was found to be induced by Polyethylene Glycol (PEG) 6000 and exogenous ABA treatment. Overexpression of *AhDREB1* in *Arabidopsis* increased ABA sensitivity during seed germination, affected the expression of ABA signaling pathway-related genes and enhanced drought resistance. Furthermore, the H3ac enrichment level was increased in various regions of *AhDREB1* chromatin during osmotic stress and treatment with the specific HDAC inhibitor trichostatin A (TSA). In summary, histone acetylation can affect the expression of *AhDREB1* under drought conditions, and may be important in further improving drought resistance of peanut plants.

## 2. Results

### 2.1. Isolation and Characterization of Peanut AhDREB1 Gene

**Figure 1:**
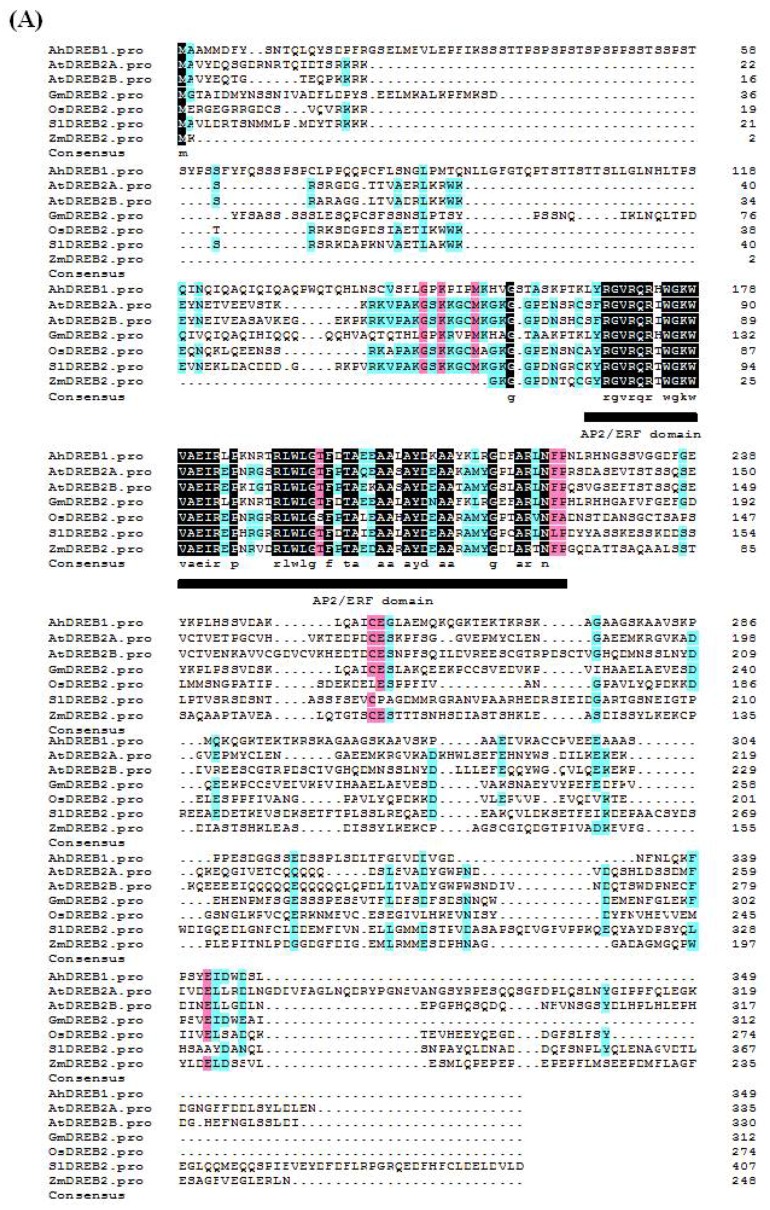

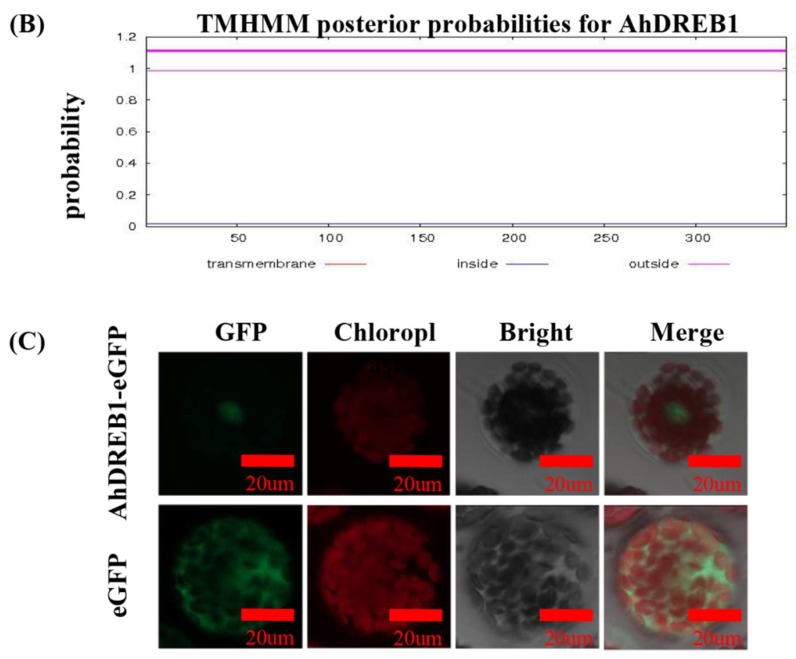
AhDREB1 is a member of the APETALA2/Ethylene Responsive Factor (AP2/ERF) transcription factor family in peanut. (**A**) Alignment of amino acid sequences of AhDREB1 with six dehydration-responsive element binding protein (DREB) subfamily proteins. AhDREB1: GenBank number API65088.1; AtDREB2A: GenBank number BAA33794.1; AtDREB2B: GenBank number BAA33795.1; GmDREB2: GenBank number AAQ57226.1; OsDREB2: GenBank number AAN02487.2; SlDREB2: GenBank number ADZ15315.1; ZmDREB2: GenBank number AFI71287.1. Identical amino acid residues are shaded in black. Conservative AP2/ERF domain are marked with a black underline. (**B**) AhDREB1 has no transmembrane domain, transmembrane structure was analyzed using online TMHMM 2.0 (http://www.cbs.dtu.dk/services/). (**C**) The intracellular localization of AhDREB1 in *Arabidopsis* protoplasts. Image showed the eGFP protein was observed in the whole *Arabidopsis* protoplasts cells, which the AhDREB1-eGFP fusion protein was localized in nucleus in *Arabidopsis* protoplasts cells, determined by confocal and bright-field microscopy.

The full-length CDS of *AhDREB1* gene was isolated from peanut leaf DNA consists of a 1050-bp open reading frame (ORF) that encodes a polypeptide of 349 amino acid residues and predicted molecular mass ~37.8 kDa (Figure 1A). Sequence analysis showed that AhDREB1 contains one conserved AP2/ERF DBD (residues 167–227), including the two conserved residues V14 and L19 specific to DREB TFs [18]. Multiple sequence alignment using DNAMAN8.0 software suggested that *AhDREB1* also has a low degree of similarity to the known *DREB* genes from *A. thaliana*, *Glycine max*, *Oryza sativa*, *Solanum lycopersicum* and *Zea mays* outside the AP2/ERF domain (Supplementary Figure S1). The *AhDREB1* promoter sequence contains many putative stress response-related *cis*-elements, including examples of the ABRE element (6 hits), CE3 recognition site (1 hit), MYB recognition site (1 hit) and heat shock response element (HSE) recognition site (3 hits) (Supplementary Figure S2). Secondary structure prediction suggested AhDREB1 has no transmembrane structure (Figure 1B). To determine the subcellular localization of AhDREB1, we engineered a plasmid construct in which the *AhDREB1* sequence without the stop codon was inserted into the 5′ end of the *eGFP* gene in the p35S-eGFP vector. The p35S::AhDREB1-eGFP recombinant plasmid was transformed into *Arabidopsis* protoplast cells, with the empty p35s-eGFP vector used as a control. Confocal microscopy imaging showed that, while the eGFP (enhanced Green Fluorescent Protein) control protein was distributed throughout the cell, the AhDREB1-eGFP fusion protein was only visible in the nucleus (Figure 1C). These results suggest that *AhDREB1* encodes a nuclear protein containing one conserved AP2/ERF DBD, and that AhDREB1 belongs to the DREB TF subfamily.

### 2.2. Expression of AhDREB1 Is Induced by Osmotic Stress and ABA Treatment

**Figure 2:**
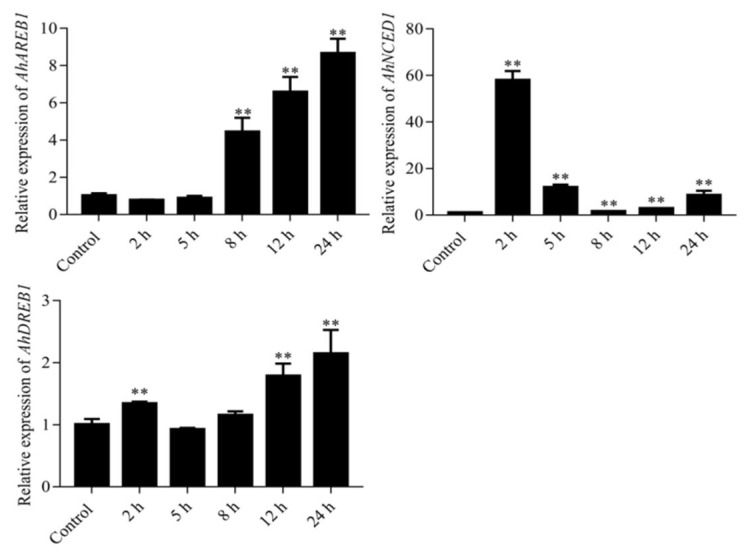
Expression analyses of *AhDREB1*, *AhNCED1* and *AhAREB1* following 20% PEG treatment in peanut leaves by real-time quantitative PCR (RT-qPCR). Time points of 2, 5, 8, 12, and 24 h were sampled to observe the expression changing trend. The untreated group was used as the control. Mean and SD were obtained from more than three biological replicates. Asterisks indicate significant differences from control (Student’s *t* test *p* values, ** *p* < 0.01).

**Figure 3:**
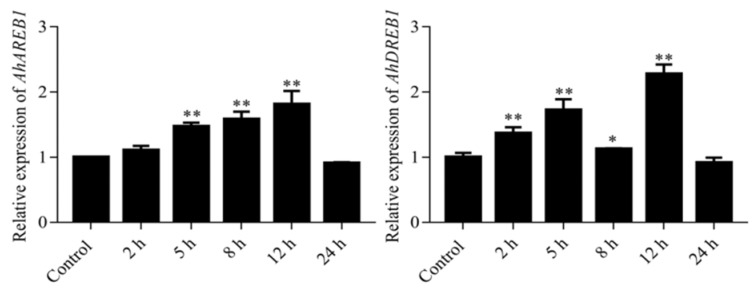
Expression analyses of *AhDREB1* and *AhAREB1* following 100 μM abscisic acid (ABA) treatment in peanut leaves by RT-qPCR. Time points of 2, 5, 8, 12, and 24 h were sampled to observe the expression changing trend. The untreated group was used as the control. Mean and SD were obtained from more than three biological replicates. Asterisks indicate significant differences from control (Student’s *t* test *p* values, * *p* < 0.05 and ** *p* < 0.01).

The expression profile of *AhDREB1* was examined in peanut leaves treated with 20% PEG 6000 or 100 μM ABA. *AhDREB1* expression was increased at 2 h of PEG treatment, slightly declined at 5 h, then significantly increased and maintained a relatively high level from 8 to 24 h. The drought-related genes *AhAREB1* and *AhNCED1* were also expressed in response to PEG. *AhAREB1* levels did not change significantly at time points up to 5 h, but then were significantly upregulated at 8 h, and continuously increased from 12 to 24 h. The expression of *AhNCED1* was significantly increased at 2 h, slightly decreased at 5 to 8 h, although it remained higher than the control level, then slowly increased from 12 to 24 h (Figure 2). The expression of *AhDREB1* could be induced by osmotic stress caused by PEG treatment suggests that *AhDREB1* itself is a drought-responsive gene. Consistent with this, we found that 100 μM ABA treatment produced a remarkable increase in *AhDREB1* expression level at 2 to 5 h, which slightly declined at 8 h, then significantly increased at 12 h, and finally declined again at 24 h. In comparison, *AhAREB1* expression continuously increased from 2 to 12 h, and then declined 24 h after ABA treatment (Figure 3). Thus, *AhDREB1* expression can be increased by both osmotic stress and treatment with exogenous ABA.

### 2.3. Overexpression of AhDREB1 in Arabidopsis Enhances Plant Tolerance to Drought Stress

**Figure 4:**
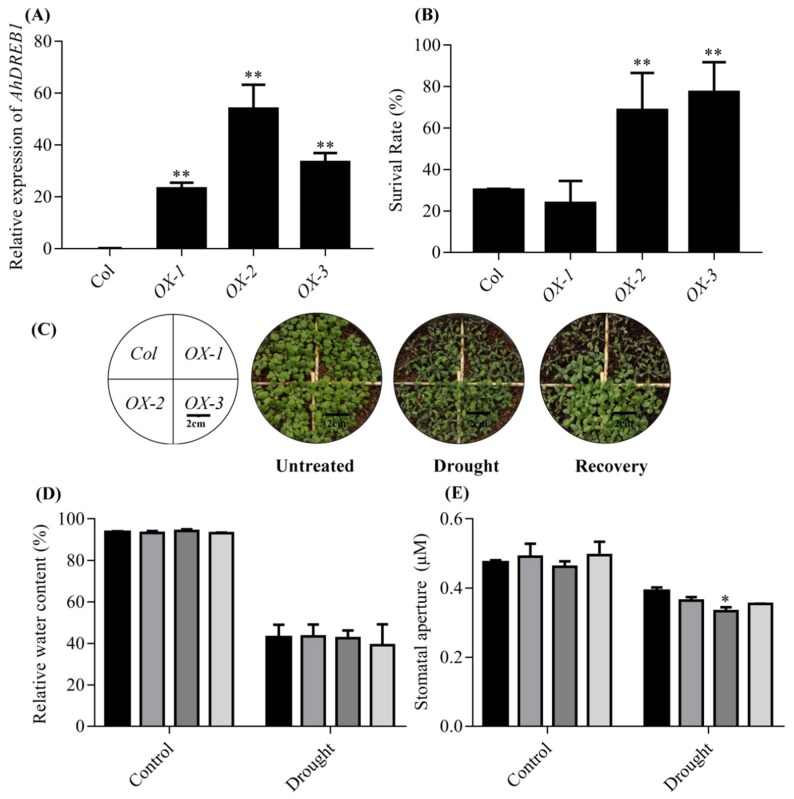

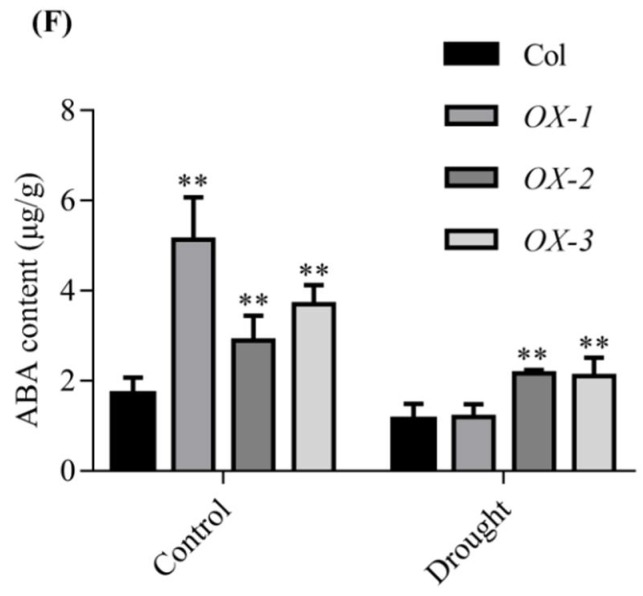
Overexpression of *AhDREB1* in *Arabidopsis* enhances plant tolerance to drought stress. (**A**) The expression levels of *AhDREB1* in *AhDREB1-OX* lines. (**B**) Survival rate of 3-week-old wild-type and *AhDREB1-OX* lines during the drought stress test. (**C**) Drought tolerance phenotype of 3-week-old wild-type and *AhDREB1-OX* lines were drought stress for 2 weeks and rehydration 2days. (**D**) The change in water content in overexpression *Arabidopsis thaliana*. (**E**) Stomatal opening in the leaves of 3-week-old wild-type and *AhDREB1-OX* lines under control conditions or after drought stress for 2 weeks; *n* = 180. ‘*’ indicates a significant difference at the level of *p* < 0.05 between *AhDREB1-OX* lines and Col plants under control treatment or drought stress conditions. (**F**) ABA content in the leaves of 3-week-old wild-type and *AhDREB1-OX* lines under control conditions or after drought stress for 2 weeks. All experiments, mean and SD were obtained from more than three biological replicates. Asterisks in (**A**) to (**F**), indicate significant differences from Col (Student’s *t* test *P* values, * *p* < 0.05 and ** *p* < 0.01).

*AhDREB1* overexpression (*AhDREB1-OX*) *Arabidopsis* lines were obtained to understand the role of *AhDREB1* in the drought stress response. Of more than 20 independent lines produced, three independent T_3_ homozygous *AhDREB1-OX* lines (*OX-1*, *OX-2*, *OX-3*) with a relatively high level of *AhDREB1* transcription were selected for further studies (Figure 4A). We first examined whether *AhDREB1-OX* lines at the seedling stage display tolerant phenotypes under drought stress conditions. When three-week-old Col and *AhDREB1-OX* lines were drought stressed for two weeks, *OX-2* and *OX-3* plants exhibited strong drought tolerance, while Col and *OX-1* plants showed severe wilting (Figure 4C). After 2 d rehydration, almost all wild-type and *OX-1* plants failed to recover, while *OX-2* and *OX-3* plants recovered rapidly (Figure 4C). After recovery, 68% of *OX-2* and 77% of *OX-3* plants survived, in contrast to only 30% of Col and 24% of *OX-1* plants (Figure 4B). We found that water content in *AhDREB1-OX* lines was not significantly different to that of Col plants during dehydration treatment (Figure 4D), but the stomatal aperture in leaves of *OX-2* plants was smaller than in Col plants under stress conditions (Figure 4E) (Supplementary Figure S3). The endogenous ABA levels in the rosette leaves of all three independent T_3_ homozygous transgenic lines were significantly higher than Col under normal growth conditions. Intriguingly, however, ABA content of *OX-2* and *OX-3* plants was higher than that in Col plants under drought stress conditions, although *OX-1* ABA levels were not different to controls (Figure 4F).

**Figure 5:**
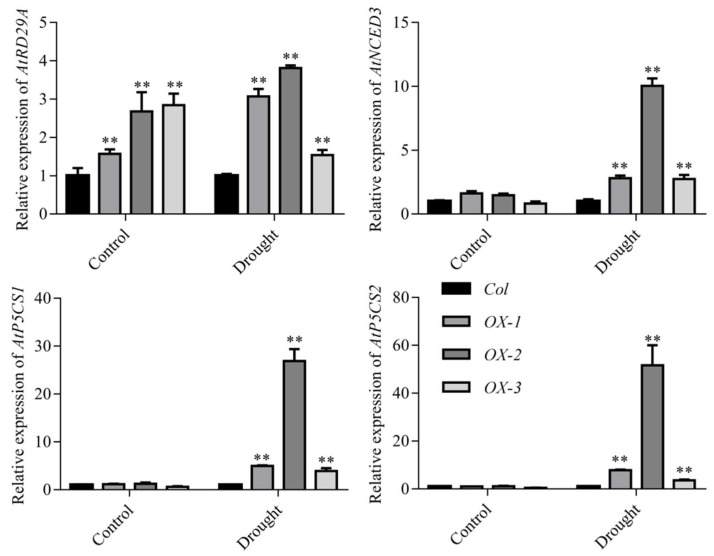
Expression analyses of drought stress-related marker genes: *AtRD29A*, *AtNCED3*, *AtP5CS1*, *AtP5CS2* in the wild type and transgenic plants under drought stress. The untreated group was used as the control. Mean and SD were obtained from more than three biological replicates. Asterisks indicate significant differences from control (Student’s *t* test *P* values, ** *p* < 0.01).

Given that *AhDREB1-OX* plants showed enhanced drought tolerance, we investigated whether the transcription of drought stress-related marker genes was altered compared to Col control plants. We found that transcript levels of the drought stress-related genes *AtRD29A*, *AtNCED3*, *AtP5CS1* and *AtP5CS2* were significantly higher than Col in all three transgenic lines after drought treatment, especially in *OX-2* plants (Figure 5). At the same time, the transcriptional level of *AhDREB1* was also significantly increased during PEG treatment. These results indicate that overexpression of *AhDREB1* can promote downstream drought-related gene expression. In summary, overexpression of *AhDREB1* can enhance drought resistance of the vegetative stages of *A. thaliana* by increasing ABA levels and promoting the expression of drought-resistance genes.

### 2.4. AhDREB1 Increases ABA Sensitivity and Affects ABA Signaling Pathway in AhDREB1-OX Arabidopsis Plants

**Figure 6:**
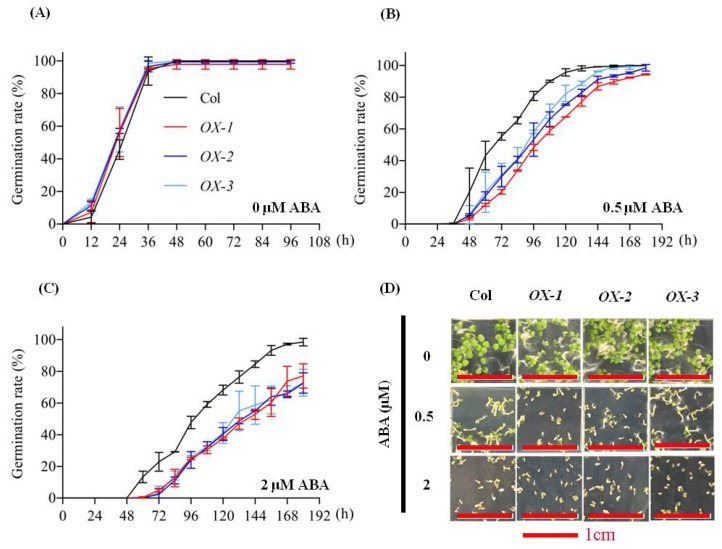
AhDREB1 increases ABA sensitivity in *Arabidopsis*. (**A**–**C**) Seed germination rate of *AhDREB1-OX* lines and Col in response to different concentrations of ABA. Numbers of germinated seedlings were recorded from 0 to 192 h after stratification on one-half-strength Murashige and Skoog (1/2 MS) agar plates containing 0, 0.5, or 2 μM ABA, respectively. (**D**) Photographs of seed germination on agar plates containing 0, 0.5 or 2 μM ABA. Mean and SD were obtained from more than three biological replicates.

**Figure 7:**
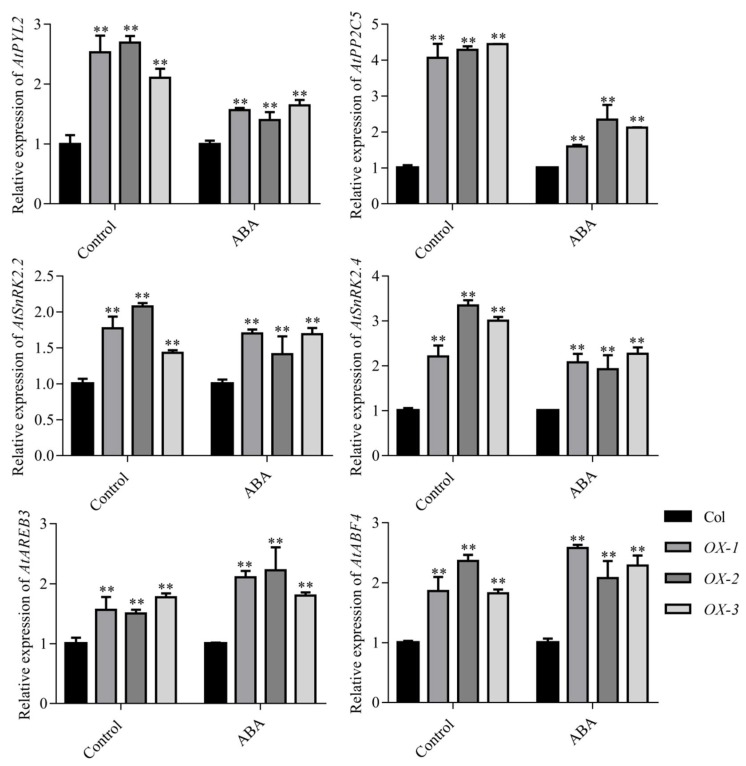
Expression changes of ABA signaling pathway related gene: *AtPYL2*, *AtPP2C5*, *AtSnRK2.2*, *AtSnRK2.4*, *AtAREB3*, *AtABF4* following 10 μM ABA treatment. The untreated group was used as the control. Mean and SD were obtained from more than three biological replicates. Asterisks indicate significant differences from control (Student’s *t* test *p* values, ** *p* < 0.01).

To investigate whether *AhDREB1* affects plant drought tolerance via the ABA-dependent pathway, we checked ABA sensitivity of *AhDREB1-OX* transgenic plants at the germination stage. Seeds of the three overexpression lines, together with Col controls, were germinated in one-half-strength Murashige and Skoog (1/2 MS) medium containing ABA at one of three different concentrations (0, 0.5, 2 μM). *AhDREB1-OX* seeds exhibited a significantly slower germination rate than wild type following 0.5 and 2 μM ABA treatment, but there were no differences between *AhDREB1-OX* and Col plants without ABA treatment (Figure 6A–D). Thus, ABA sensitivity of *AhDREB1-OX* plants was increased. Furthermore, the transcription levels of ABA signaling pathway-related genes *AtPYL2*, *AtPP2C5*, *AtSnRK2.2*, *AtSnRK2.4*, *AtAREB3* and *AtABF4* were significantly higher than in Col plants under both normal growth conditions and after exogenous application of 10 μM ABA (Figure 7). In conclusion, *AhDREB1* increases ABA sensitivity and affects the ABA signaling pathway in *AhDREB1-OX Arabidopsis* plants.

### 2.5. Histone Acetylation Is Involved in AhDREB1 Transcriptional Regulation

**Figure 8:**
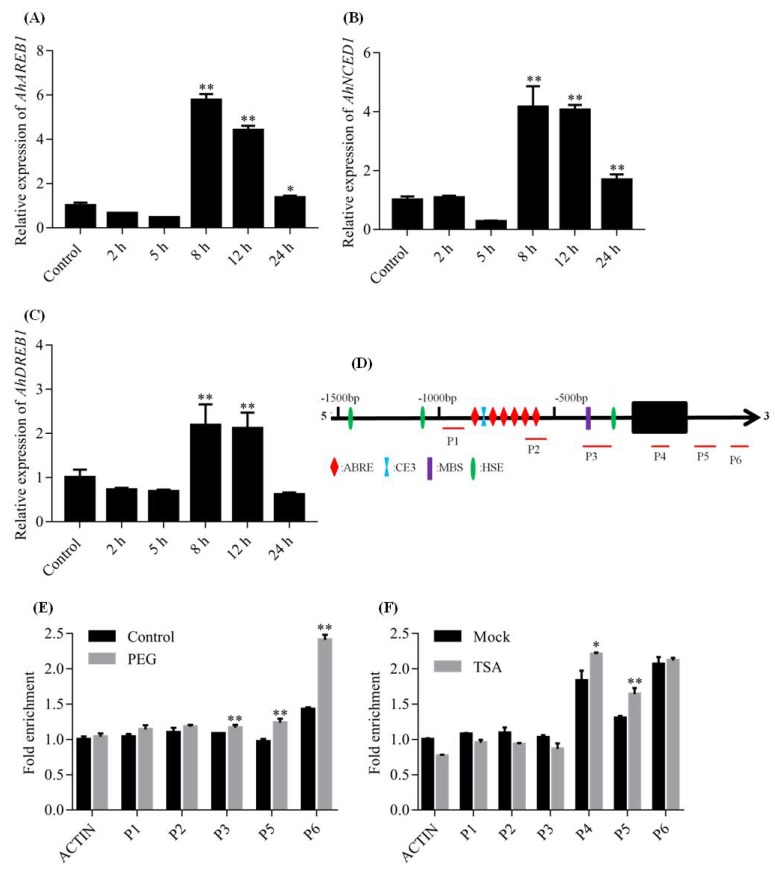
Histone acetylation is involved in *AhDREB1* transcriptional regulation. (**A**–**C**) Expression analyses of *AhDREB1* and stress resistance genes following 1 μΜ treatment in peanut leaves by RT-qPCR. Time points of 2, 5, 8, 12, and 24 h were sampled to observe the expression changing trend. The untreated group was used as the control. Mean and SD were obtained from more than three biological replicates. Asterisks indicate significant differences from control (Student’s *t* test *p* values, * *p* < 0.05 and ** *p* < 0.01). (**D**) The structure of the *AhDREB1* promoter. PCR amplification (P1–P6) for chromatin immunoprecipitation (ChIP) assays are indicated. (**E**,**F**) H3ac levels in chromatin of peanut leaves under 1 μM TSA and 20% PEG treatment, respectively. The untreated group was used as the control. Mean and SD were obtained from more than three biological replicates. Asterisks indicate significant differences from control (Student’s *t* test *p* values, * *p* < 0.05 and ** *p* < 0.01).

Trichostatin A (TSA) is a small-molecule histone deacetylase (HDAC) inhibitor, that is, it represses histone deacetylation. We found that TSA treatment of peanut leaves slightly reduced the expression of *AhDREB1* at 2 to 5 h, while a relatively high transcription level of *AhDREB1* was detected at 8 to 12 h, followed by a decrease in expression at 24 h (Figure 8A). When we checked the drought-related genes *AhAREB1* and *AhNCED1*, we observed that their expression profiles were both essentially consistent with that of *AhDREB1* during TSA treatment (Figure 8B,C). These results suggest that TSA can promote the expression of *AhDREB1* and, therefore, that histone acetylation modification may be involved in its transcriptional regulation. To clarify this, chromatin immunoprecipitation (ChIP) assays were performed using antibodies that specifically recognize histone H3ac modifications. H3ac levels in chromatin of peanut leaves treated with TSA and PEG were analyzed in various regions of the *AhDREB1* gene using ChIP-quantitative PCR (qPCR). TSA treatment resulted in relatively high H3ac enrichment levels in the P4 and P5 regions of *AhDREB1* chromatin, whereas no statistically significant changes in H3ac were detected in other regions (Figure 8E). Treatment with 20% PEG also enriched levels of H3ac, but only in the P3, P5, P6 regions (Figure 8F). In contrast, the application of 100 μM ABA (Supplementary Figure S4) produced no significant changes in H3ac levels in any region of *AhDREB1* chromatin. In conclusion, histone acetylation is involved in *AhDREB1* transcriptional regulation and the level of the histone acetylation in *AhDREB1* chromatin can be modulated by both TSA and 20% PEG treatment.

## 3. Discussion

AP2/ERFs are plant-specific transcription factors. Increasing evidence shows that AP2/ERF TF genes are induced in response to various stresses, including drought, abscisic acid, salt, cold, heat and alkalinity [42]. Here, we isolated full-length CDS of *AhDREB1* gene, which encodes an AP2/ERF TF of the DREB subfamily (Figure 1A). The expression of *AhDREB1* is induced by PEG 6000 and ABA (Figure 2 and Figure 3), which suggests that *AhDREB1* might play an important role in the plant drought response and the ABA signaling pathway. A large number of studies have revealed that AP2/ERF TFs can enhance the stress tolerance when overexpressed in transgenic plants [20,43]. For instance, overexpression of *ERF1-V* in wheat improves resistance to powdery mildew, salt and drought stress [44]. Similarly, overexpression of *SpERF1* enhances drought tolerance of transgenic *A. thaliana* [45]. It has been reported that overexpression of *OsERF71* can enhance the drought stress tolerance of rice by changing the morphological structure of roots and by regulating the expression of genes related to lignin synthesis and drought-related genes [46]. In the current paper, overexpression of AhDREB1 significantly enhanced drought resistance in A. thaliana (Figure 4C) in the vegetative stages of at least two OX lines (Figure 4B). 

It is well known that ABA is a pivotal regulator of plant responses to drought stress. The level of ABA increases rapidly under drought stress and this is a function of its biosynthesis, catabolism and transport [10]. NCED is a key enzyme in the ABA synthesis pathway [10,11] and, in *Arabidopsis*, the respective gene *AtNCED3*, which is induced by drought stress, controls the level of endogenous ABA under drought-stress conditions. Overexpression of *AtNCED3* causes an increase in endogenous ABA levels and promotes transcription of drought- and ABA-inducible genes, which improve drought tolerance in *Arabidopsis* [47]. In peanut plants, the expression of *AhNCED1* is significantly upregulated by dehydration and high salinity, while overexpression of *AhNCED1* in *Arabidopsis* plants leads to the accumulation of endogenous ABA and improved water-stress tolerance [48]. ABA transport is also an important factor affecting ABA levels and plant responses to drought stress. Previous studies have shown that mutation of the *Arabidopsis abcg40* gene, which encodes an ABA transporter, causes stomata to close more slowly in response to ABA, and strongly delays the upregulation of ABA-responsive genes, resulting in reduced drought tolerance [49]. In peanut, *AhATL1*, an ABCG transporter subfamily gene, is upregulated by water stress and exogenous ABA treatment. Overexpression of *AhATL1* decreases ABA sensitivity and drought resistance by influencing ABA transport in *Arabidopsis* [50]. These studies show that the level of ABA is closely related to drought tolerance and thus ABA levels can be used as an important index of drought resistance in plants [9]. Consistent with their drought-resistant phenotype, we showed here that ABA contents of the *Arabidopsis AhDREB1-OX* lines *OX-2* and *OX-3* were significantly higher than in Col under drought stress, although line *OX-1* showed no significant difference to the control (Figure 4F). We suspected that the phenomena may be related to the expression of *AhDREB1* in *Arabidopsis thaliana*. Indeed, *AhDREB1* expression in *OX-1* line is relatively low compared to the other two lines (Figure 4A), which may affect the level of ABA under drought stress and affect the drought resistance of *Arabidopsis*. It is interesting that the ABA levels of *AhDREB1-OX* lines were also significantly higher than Col under normal growth conditions. Previous studies have shown that ABA is an important factor in regulating seed dormancy and germination [51]. In *Arabidopsis*, strains overexpressing *AtDREB2C* are ABA hypersensitive during germination and show enhanced plant tolerance to cold and heat stress [8]. The overexpression of *GhERF38* increased ABA sensitivity in seed germination and seedling period in *Arabidopsis* [52]. Our *AhDREB1-OX* lines were also ABA hypersensitive during seed germination and seedling development. Thus we speculated that AhDREB1 may affect ABA signaling pathway in *Arabidopsis*. To verify this hypothesis, we checked expression levels of ABA signaling pathway-related genes in *AhDREB1-OX* lines and Col plants subjected to 10 μM ABA treatment. All of these genes showed significantly higher expression than the wild-type under both normal conditions and during ABA treatment (Figure 7). These results suggest *AhDREB1* increases ABA sensitivity and affects ABA signaling pathway in *Arabidopsis*.

It has been reported that ERF and DREB subfamily TFs can directly interact with GCC boxes (GCCGCC) or DRE elements in the promoters of downstream stress- and defence-related genes to regulate multiple stress responses [20]. Overexpression of *SpERF1* enhances drought tolerance of transgenic *A. thaliana* after binding to DRE elements in the promoters of the abiotic stress-responsive genes *P5CS1*, *RD29A*, *HSP101*, *LEA4-5*, *HSP70* and *COR47* [45]. ThCRF1 binds DRE and CGG-box motifs and induces the expression of various genes, including *P5CS*, *SOD* and *POD*, thereby enhancing tolerance to salt stress [17]. In this paper, to further investigate the molecular mechanism by which *AhDREB1* enhances drought tolerance, RT-qPCR was carried out to quantify transcription levels of *RD29A*, *P5CS1*, *P5CS2* and *NCED1*. All four genes were significantly upregulated in *AhDREB1-OX* lines subjected to drought stress (Figure 5). This result was consistent with the drought-resistant phenotype described above. In summary, AhDREB1 affects ABA sensitivity and ABA signaling by increasing the level of endogenous ABA and the expression of downstream drought stress-related genes, which in turn increase *A. thaliana* drought resistance in overexpressing lines.

Histone acetylation is one of the most important histone modifications and can affect chromatin structure and gene expression. TSA, a small molecule HDAC inhibitor, can cause transient increases in acetylation of histone H2B, H4, and H3 in chromatin [53]. It has been reported that *RAB18*, *RD29B* and *HSP70*, together with four late embryogenesis abundant protein (*LEA*) genes, are upregulated by TSA during seed germination [54]. TSA also suppresses cold-induced transcription of the *ZmDREB1* gene in maize [39]. In our studies, the expression of *AhDREB1* was significantly upregulated by TSA (Figure 8A), implying that histone acetylation may be involved in the transcriptional regulation of *AhDREB1*. We investigated the effect of TSA treatment on the enrichment level of H3ac in *AhDREB1* chromatin using ChIP-qPCR assays. The levels of H3ac were significantly increased in the P4 and P5 regions of *AhDREB1* (Figure 8E), consistent with our *AhDREB1* gene expression results following TSA treatment. We therefore conclude that histone acetylation is involved in the transcriptional regulation of *AhDREB1*. 

Many studies have shown that histone acetylation plays an important role in the regulation of plant growth and development, and in the responses to abiotic stress. Thus, in *Arabidopsis*, H3K4me3 and H3K9ac levels increase in the coding regions of *RD29A*, *RD29B*, *RD20* and *RAP2.4* genes during drought stress [55]. HDACs positively affect the expression of the cold-induced *ZmDREB1* gene, and this affects plant tolerance to cold stress [39]. Acetylation of H3K9 and K3K14 is increased under PEG and ABA treatment in *A. hypogaea* [36], while in *A. thaliana*, AtHDA9 negatively affects plant sensitivity to drought and salt stresses by modulating histone acetylation levels of a large number of stress-responsive genes [27]. In this paper, we found that overexpression of *AhDREB1* enhances plant tolerance to drought stress and that *AhDREB1* transcription is affected by histone acetylation. Accordingly, we hypothesised that *AhDREB1* is affected by histone acetylation under drought conditions, and that this affects drought resistance in whole plants. To verify the hypothesis, we tested H3ac levels following treatment with 20% PEG and found these to increase in the P3, P5, P6 regions of the *AhDREB1* gene (Figure 8F). However, treatment with 100 μM ABA yielded no significant change in H3ac levels (Supplementary Figure S4). In conclusion, histone acetylation is involved in the transcriptional regulation of *AhDREB1* under osmotic stress conditions by increasing H3ac levels, thereby further improving plant drought resistance. 

In summary, we isolated a full-length CDS of the AP2/ERF family gene *AhDREB1* from peanut leaves and showed that *AhDREB1* overexpression can improve the tolerance of *Arabidopsis* to drought via the ABA-dependent pathway. At the same time, histone acetylation is involved in the transcriptional regulation of *AhDREB1* during osmotic stress, further improving drought resistance. This is the first report that histone acetylation is involved in the transcriptional regulation of a *DREB* subfamily gene in peanut. The findings provide new insight into how AP2/ERF TFs enhance plant tolerance to drought stress, which may lead to successful breeding of drought resistant strains of peanuts and other crops.

## 4. Material and Methods

### 4.1. Peanut Plant Material and Growth Conditions

Seeds of peanut (*Arachis hypogaea* L. cv. Yueyou 7) were provided by the Crop Research Institute, Guangdong Academy of Agricultural Sciences, China. Seeds were soaked in water for 12 h, and then transferred to moist filter paper in an artificial climate incubator with a cycle of 16 h light from fluorescent and incandescent lamps (200 μmol·m^−2^·s^−1^) at 26 °C followed by 8 h darkness at 22 °C for 48 h until germination. Germinated seeds were planted in a potting mixture of vermiculite, perlite and soil (1:1:1) in the artificial climate incubator. Plants were watered with half-strength Murashige and Skoog nutrient solution every other day [36].

### 4.2. Isolation and Sequence Analysis of AhDREB1 from Arachis hypogaea L.

The total RNA of peanut leaves was extracted. Reverse transcription is performed by using 200 units Superscript III Reverse Transcriptase (Invitrogen, catalog no. 18080, Shanghai, China) and 500 ng oligo-dT primer and then the first strand of cDNA was synthesized. Reverse transcribed (RT) conditions were: 70 °C for 10 min (min), 42 °C for 1 h, and 15 min at 70 °C [36]. The cDNA was used as a template to amplify *AhDREB1* open reading frame (ORF) by PCR using the following primers: *AhDREB1* ORF forward primer 5′-ATGGCAGCAATGATGGATTTCTACA-3′, *AhDREB1* ORF reverse primer 5′-TCACAGAGAATCCCAATCAATCTCA-3′. PCR amplification was performed as follows: 94 °C for 7 min, then 35 cycles of 94 °C for 30 s (s), 55 °C for 30 s and 72 °C for 1 min 30 s, with a final extension step at 72 °C for 10 min. PCR products were purified with an Agarose Gel DNA Purification Kit (TaKaRa, catalog no. DV805A, Dalian, China) and were ligated into the pMD19 T-vector (TaKaRa, catalog no. 6013, Dalian, China). Plasmids were sequenced on both strands.

Sequence analysis was performed using Lasergene 7.0 software (DNAStar, Madison, WI, USA). On-line BLAST analysis of DNA and amino acid sequences was performed at the National Center for Biotechnology Information Services website (https://blast.ncbi.nlm.nih.gov/Blast.cgi). Multiple sequence alignment was performed using DNAMAN8.0 software [56]. The *AhDREB1* promoter sequence was identified using the PlantCARE database (http://bioinformatics.psb.ugent.be/webtools/plantcare/html/). AhDREB1 was assessed for the presence of a transmembrane domain using online TMHMM 2.0 (http://www.cbs.dtu.dk/services/) [50]. Promoter elements were analyzed using PlantCARE [57,58].

### 4.3. Subcellular Localization Analysis

The expression vectors for subcellular localization were constructed as follows: the *AhDREB1* coding region was prepared by PCR using the primer set forward primer 5′-ATGGCAGCAATGATGGATTTCTACA-3′ and reverse primer 5′-TCACAGAGAATCCCAATCAATCTCA-3′. The amplified fragment was cloned into pMD19 T-vector and sequenced on both strands. Subsequently, the *AhDREB1* ORF was cloned into the p35S::eGFP vector [59]. The fusion construct p35S::AhDREB1-eGFP was driven by the 35S promoter. The recombinant plasmid vector was transformed into *Arabidopsis* protoplasts. After incubation for 12–18 h in the dark, eGFP fluorescence signals in protoplasts were observed under the confocal microscope (Carl Zeiss LSM 710; Carl Zeiss, Germany).

### 4.4. Peanut Plant Treatments

Peanut seedlings were removed from the soil at the four-leaf stage, and after the roots were carefully rinsed with deionized water, they were maintained in 1/8 MS medium for up to 2 h. Seedlings were treated with 20% PEG or 100 μM ABA for 2, 5, 8, 12 and 24 h. Trichostatin A (TSA) was used to treat seedlings at 1 μM for 2, 5, 8, 12 and 24 h [36]. Untreated seedlings planted in the soil were used as control plants. Seedlings placed in an equivalent volume of deionized water instead of ABA or TSA solutions were used as mock treatments. Seedlings were transferred to an artificial climate incubator (26 °C, 60% moisture) under continuous light. Peanut leaves were harvested and frozen at −80 °C prior to use.

### 4.5. Real-Time Quantitative PCR (RT-qPCR)

Total RNA was extracted from peanut leaves or *Arabidopsis thaliana* seedlings [36]. For first-strand cDNA synthesis, 1 μg high-quality total RNA was reverse transcribed (RT) using a Prime Script TM RT Reagent Kit (Perfect Real Time, TaKaRa, Dalian, China). The cDNA sample was used as the template for RT-qPCR. Relative transcript levels of *AhDREB1* were evaluated in an ABI 7500 system using the ChamQ SYBR qPCR Master Mix (Low ROX Premixed, Vazyme Biotech Co., Ltd., Nanjing, China). Each reaction contained 5 μL 2 × ChamQ SYBR qPCR Master Mix, 10 ng cDNA, 0.2 μL 0.05 mM stock solution of each primer in a final volume of 10 μL. The PCR thermal cycles were as follows: 95 °C for 30 s, followed by 40 cycles at 95 °C for 5 s and 60 °C for 34 s. The *AhACTIN* and *AtACTIN2* genes were used as internal controls for *A. hypogaea* and *A. thaliana*, respectively, as described [36,59]. Relative expression levels were calculated using the relative 2^−ΔΔ*C*t^ method [60]. The relevant primers for the analysis of gene transcription levels are listed one by one in Supplementary Table S1.

### 4.6. Plant Transformation and Growth Conditions

The recombinant vector p35S::AhDREB1-eGFP was transformed into *Agrobaterium tumefaciens* strain EHA105. Positive *A. tumefaciens* colonies containing the vector were employed for *Arabidopsis* Col transformation by the floral dip method [59]. T_0_ generation seeds were harvested and sowed on 1/2 MS medium containing 50 mg/L kanamycin for selection, with repeat screening until T_3_. Individual plants were genotyped and homozygous plants were selected by PCR. Subsequent experiments were perform using T_3_ seeds. *Arabidopsis* growing conditions: light (a daily cycle of 16 h light and 8 h dark), temperature (22 ± 2 °C), relative humidity (60–70%).

### 4.7. Drought Stress Treatment of Arabidopsis

The seeds of *AhDREB1* overexpression lines and wild type *Arabidopsis* were grown in 1/2 MS medium and were vernalized for 48 h at 4 °C. Then seeds were placed in a greenhouse with a daily cycle of 16 h light and 8 h dark at 20 ± 2 °C. After germination, the seedlings were planted in peat soil and grown for three weeks under the same light and temperature conditions. Three-week-old *Arabidopsis thaliana* was treated by soil drought for approximately 2 weeks under the same greenhouse conditions as above. The survival rate of *Arabidopsis thaliana* was counted after 2 days of rehydration. The survival rate was determined in three independent experiments [60].

### 4.8. Stomatal Movement Assay

To observe the stomatal opening of wild-type *A. thaliana* and *AhDREB1-OX* lines under normal growth and soil drought conditions, rosette leaves were detached from three-week-old plants. The detached rosette leaves were soaked in open stomatal buffer (10 mM MES, 5 mM KCl, 50 mM CaCl_2_, pH 6.15) in a growth chamber under incandescent lamps (200 μmol·m^−2^·s^−1^) at 20 °C for 3 h. Stomatal apertures were measured as described previously [49]. Digimizer software was used to measure stomatal apertures.

### 4.9. Relative Water Content Determination

*Arabidopsis* rosette leaves under normal growth and drought stress conditions were cut and collected to determine the fresh weight (*m_f_*). Leaf material was placed at 105 °C for 0.5 h, then dried to constant weight at 80 °C, giving the dry weight (*m_d_*). Water content (%) = (*m_f_* − *m_d_*)/*m_f_* × 100%. The relative water content was determined in triplicate for each sample [61].

### 4.10. Determination of ABA Content

As previously described, ABA was extracted from *Arabidopsis* rosette leaves under soil drought or control conditions [48]. Extraction in 80% (*v*/*v*) aqueous methanol, and high performance liquid chromatography fractionation in a SinoChrom ODS AP C18 column (250 × 4.6 mm, 5 μm, Dalian, China), were conducted as reported previously [62]. The level of ABA was determined in triplicate for each sample.

### 4.11. ABA Sensitivity

Sensitivity of *AhDREB1-OX* and wild-type seeds to ABA during seeds germination was assessed. To test the effects of ABA on germination, the seeds (80 seeds each, three repeats) were germinated on 1/2 MS medium with 0.8% agar containing a gradient concentration of ABA (0, 0.5, 2 μM), and the germination rate of the treated seeds was calculated every 12 h until 192 h [59].

### 4.12. ChIP-Quantitative PCR

For the ChIP assay, leaves (500 mg) of four-leaf-stage (two weeks after planting) peanut plants were fixed with cold MC buffer (10 mM potassium phosphate pH 7.0, 50 mM NaCl, 0.1 M sucrose, 1% formaldehyde) for 20 min by vacuum concentration. Then add glycine to make its final concentration of 125 mM, vacuum incubation 10 min to terminate cross-linking. After washing leaves in MC buffer, leaves were collected and frozen in liquid nitrogen, and stored at −80 °C. The ChIP assay was performed as described previously [63]. One microgram of anti-H3ac and rabbit IgG (Millipore, Shanghai, China) were used for immunoprecipitation. Specifically, precipitated DNA was recovered and analyzed by ChIP-qPCR using SYBR Premix ExTaq Mix (Takara Bio, Dalian, China). Peanut *ACTIN* genes were used to calculate the relative fold-enrichment of target DNA fragments. The ChIP-qPCR primers are listed in Supplemental Table S2.

### 4.13. Statistical Analysis 

Quantitative data were expressed as mean ± SD. The statistical significance of experimental data was assessed by Student *t*-test or ANOVA (one-way analysis of variance with a Least Significant Difference (LSD) post-hoc test), as appropriate, using the SPSS17.0 statistical package (Chicago, IL, USA).

## Supplementary Materials

Supplementary materials can be found at http://www.mdpi.com/1422-0067/19/5/1441/s1.
