# Supplementary material for: Expression of AhDREB1, an AP2/ERF Transcription Factor Gene from Peanut, Is Affected by Histone Acetylation and Increases Abscisic Acid Sensitivity and Tolerance to Osmotic Stress in Arabidopsis
Source: Int J Mol Sci. 2018 May 11;19(5):1441. doi: 10.3390/ijms19051441 (PMC5983730; doi:10.3390/ijms19051441)
Supplement: Supplementary file 1 [file ijms-19-01441-s001.pdf]

International Journal of Molecular Sciences

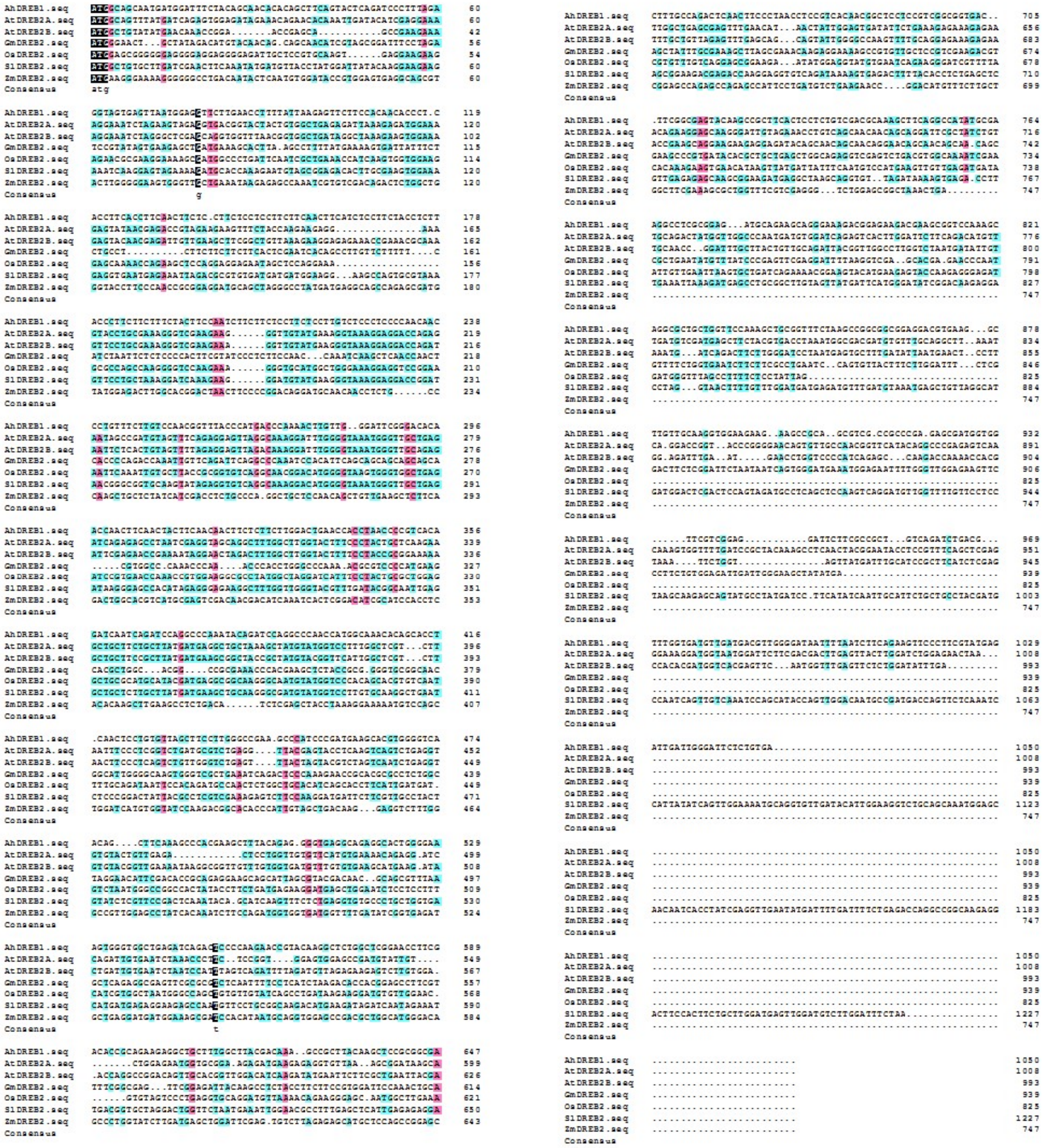

Figure S1. Alignment of sequences of *AhDREB1* with six *DREB* subfamily genes.

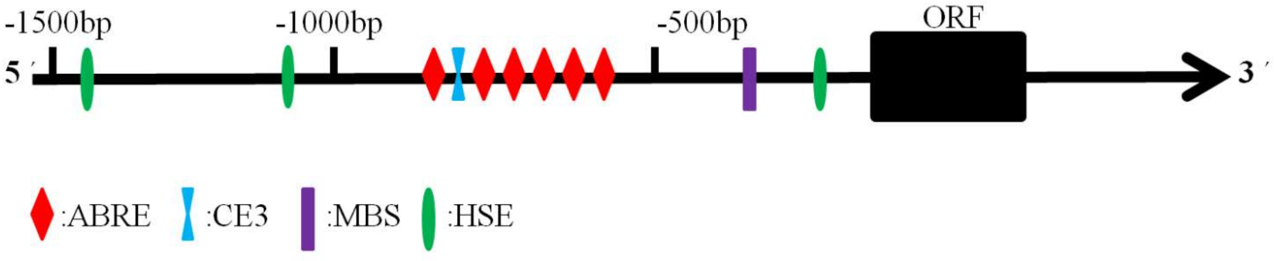

Figure S2. Analysis of cis-acting element of *AhDREB1* promoter.

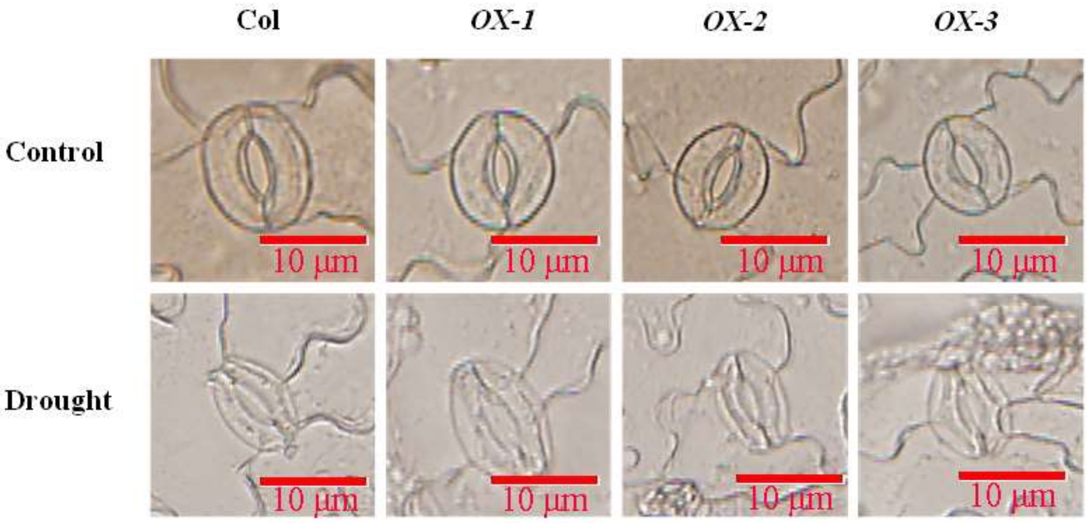

Figure S3. Images (bright-field microscopy) of stomatal apertures in the leaves of three-week-old *AhDREB1-OX* and Col plants under control or dehydration conditions.

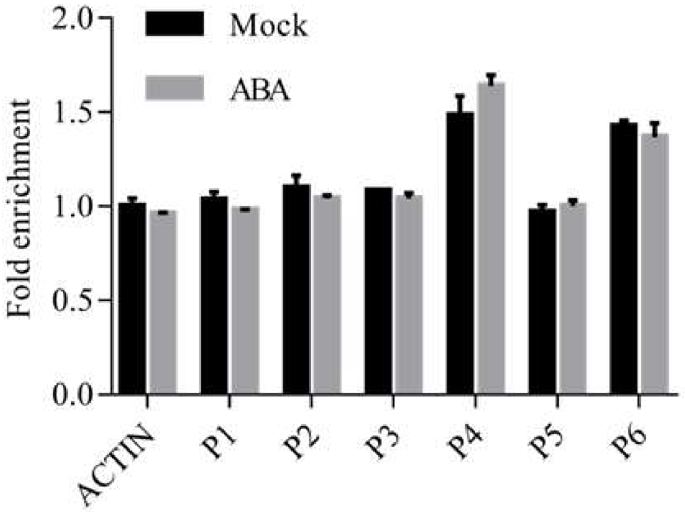

Figure S4. H3ac levels in chromatin of peanut leaves under 100µM ABA treatment.

Table S1. Primers for RT-qPCR.

Genes	Primers(5'- 3')
AhACTIN	F: GATTGGAATGGAAGCTGCTG<br>R: CGGTCAGCAATACCAGGGAA
AtACTIN2	F: GGTAACATTGTGCTCAGTGGTGG<br>R: AACGACCTTAATCTTCATGCTGC
AhAREB1	F: ACAAGGGAACCAGCATTAGG<br>R: TCACCACCACCATACCAACCA
AhNCED1	F: TTACCTGTGGGATTGTTTGC<br>R: ACATGAGCCTCTACTTCTGC
AhDREB1	F: TGGCAAACACAGCACCTCAACTC<br>R: TCTGCCTCACCCCTCTGTAAAGC
AtRD29A	F: CAGGCGTAACAGGTAAACCTAGA<br>R: TCGGAAGACACGACAGGAAACAC
AtNCED3	F: TCGCAAGATTCGGGATTTTAGAC<br>R: TTTCTGGCTCTTCCCAAGCGTTC
AtP5CS1	F: AGAGCAATGGAGTCACTTTGTATG<br>R: CCTTGGCACAGTACTCATGGTTG
AtP5CS2	F: ATTCCTCCGCCAAGTGGACAGTG<br>R: CTCAGCACCAAGTCCGAACCTAA
AtPYL2	F: CCCGAGGGAAACACAGAGGAAGA<br>R: ATAGGTGCAGATGTGGCGGCAAC

AtPP2C5	F: CGACGACGGAGGTTACAAAAATG
	R: TTGTTCCCTAGATTCATCGCAGC
AtSnRK2.2	F: TGGTCTTGCGGTGTAACATTATATG
	R:CGAGTATGTGACACTAAGGATTCTTTG
AtSnRK2.4	F: TGGCTGATGTATGGTCTTGTGGT
	R: GTTTTCCTGAAGTTCTTGGGGTC
AtAREB3	F: GAGGAGGGATAAGCAGCCTACAC
	R: ACCACCAACAGGACCATCATGGT
AtABF4	F: CAAGGATGGTAATATGGAAGGTAG
	R: GTGTCATTTCCCCTAAAGTCTGT

Table S2. Primers for ChIP-qPCR.

Fragments	Primers(5'- 3')
P1	F: CGGTTTGCTGCGTCTATTAG<br>R: CCATATCGATGCGCGTTTAC
P2	F: GAACAATGTTGACGAATCTAACCC<br>R: GCAATACTACGAGCTTCAATACC
P3	F: TTCTTTTCCCCCCTCTGTTACAC<br>R: ACTAAGCCTGGATCTCGATTCTG
P4	F: TGGCAAACACAGCACCTCAACTC<br>R: TCTGCCTCACCCCTCTGTAAAGC
P5	F: GCCCTTCCATAGATCTCATT<br>R: ATAAAAGCCAGGTCAAGCAG
P6	F: GAACAATGTTGACGAATCTAACCC<br>R: GCAATACTACGAGCTTCAATACC
AhACTIN	F: GATTGGAATGGAAGCTGCTG<br>R: CGGTCAGCAATACCAGGGAA
